# Supplementary material for: Evaluation of Effective Mass in InGaAsN/GaAs Quantum Wells Using Transient Spectroscopy
Source: Materials (Basel). 2022 Oct 30;15(21):7621. doi: 10.3390/ma15217621 (PMC9658958; doi:10.3390/ma15217621)
Supplement: Supplementary file 1 [file materials-15-07621-s001.zip › materials-1986519-supplementary.pdf]

*materials*

MDPI

*Supplementary material*

# Evaluation of effective mass in InGaAsN/GaAs quantum wells using transient spectroscopy

**Lubica Stuchlikova [1], Beata Sciana [2], Arpad Kosa [1], Matej Matus [1], Peter Benko [1], Juraj Marek [1], Martin Donoval [1], Wojciech Dawidowski [2], Damian Radziewicz [2] and Martin Weis [1,*]**

[1] Institute of Electronics and Photonics, Slovak University of Technology in Bratislava, Ilkovicova 3, 81219 Bratislava, Slovakia

[2] Faculty of Microsystem Electronics and Photonics, Wrocław University of Technology, 11/17 Janiszewskiego St., 50-372 Wrocław, Poland

* Correspondence: martin.weis@stuba.sk

### S1. Indium and nitrogen influence on the energy band offset of InGaAsN

According to reported energy band offsets (see Galluppi, M.; Geelhaar, L.; Riechert, H.; Nitrogen and indium dependence of the band offsets in InGaAsN quantum wells. Appl. Phys. Lett. 2005 86, 131925), the linear superposition of indium and nitrogen influence was assumed to be correct in narrow concentration region. As a result, the map of the energy band offsets for conduction and valence bands was reproduced as shown in Figure S1(a) and (b).

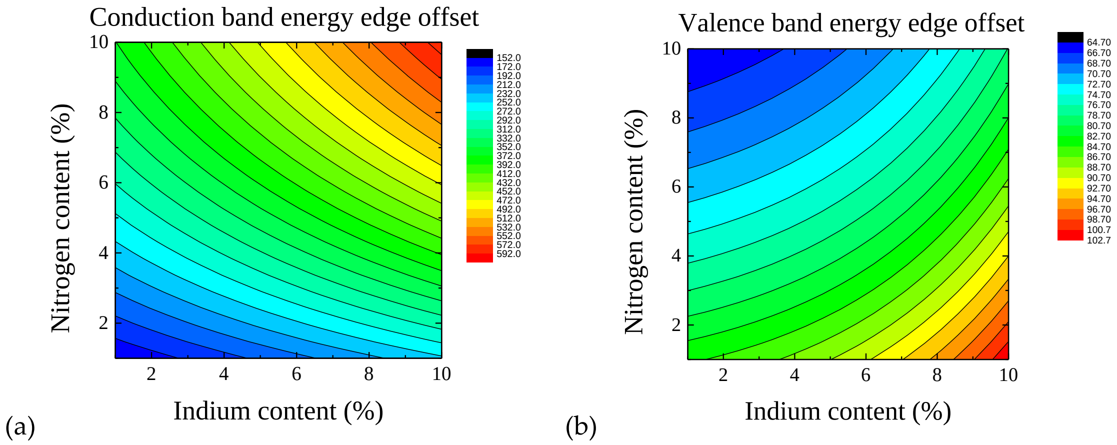


**Figure S1.** The energy band offsets for edges of (**a**) the conduction band and (**b**) the valence band.

**S2. Quantum well layer thickness estimation**

High-resolution x-ray diffractograms (HRXRD) were used to evaluate the quantum well thicknesses. Here follows HRXRD of investigated heterostructures.

**NI49n**

3 x InGaAsN/GaAs QW **In – 14,2%;** **N – 0,63%**

$d_{QW}$ – 15,5 nm

$d_{bariera}$ GaAs – 30 nm $Eg_{CER}$ = 1,1799 eV, $Eg_{BAC}$ = 1,1790 eV

$d_{cap}$ – 44 nm

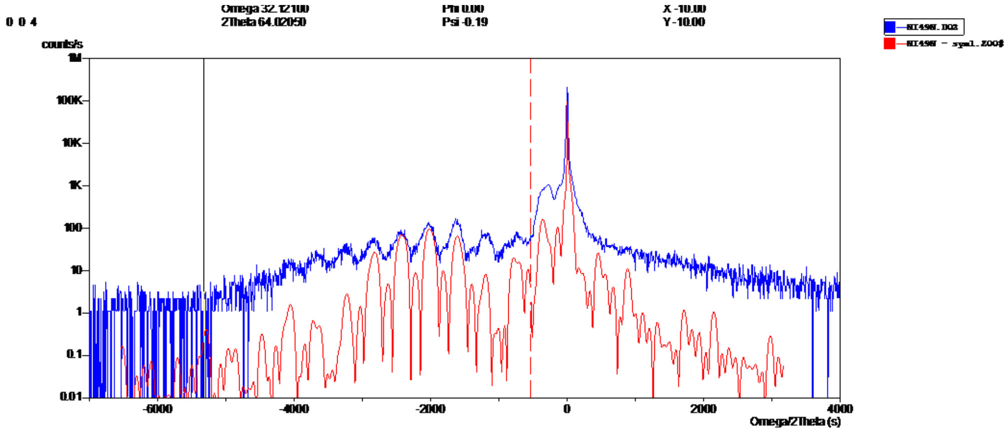


**NI51n**

3 x InGaAsN/GaAs QW **In – 14,0%;** **N – 0,44%**

$d_{QW}$ – 16,8 nm

$d_{bariera}$ GaAs – 27,5 nm $Eg_{CER}$ = 1,207 eV, $Eg_{BAC}$ = 1,204 eV

$d_{cap}$ – 40,0 nm

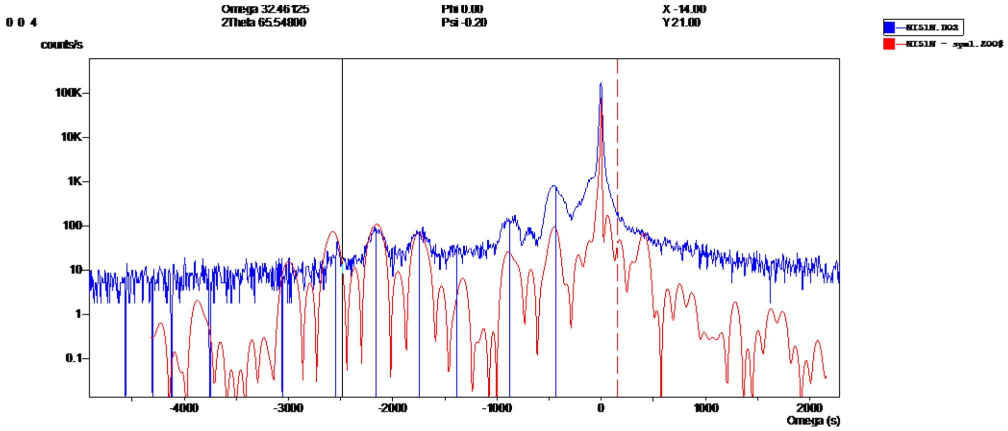


**NI55n**

3 x InGaAsN/GaAs QW **In – 15,2%;** **N – 0,42%**

$d_{QW}$ – 12,6 nm

$d_{bariera}$ GaAs – 22 nm $Eg_{CER}$ = 1,1998 eV, $Eg_{BAC}$ = 1,200 eV

$d_{cap}$ – 70 nm

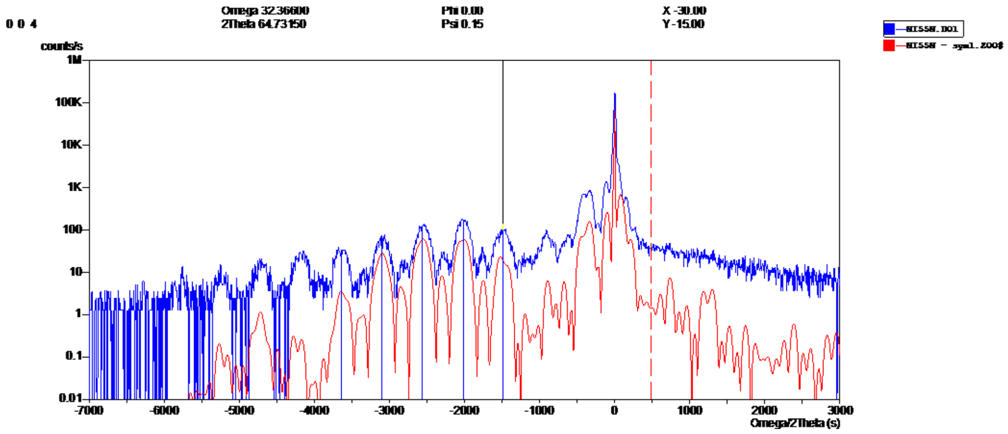


**NI57n**

3 x InGaAsN/GaAs QW **In – 3,4%; N – 0,39%**

$d_{QW}$ – 11,2 nm

$d_{bariera}$ GaAs – 24,0 nm $Eg_{CER}$ = 1,2577 eV, $Eg_{BAC}$ = 1,2577

$d_{cap}$ – 30 nm

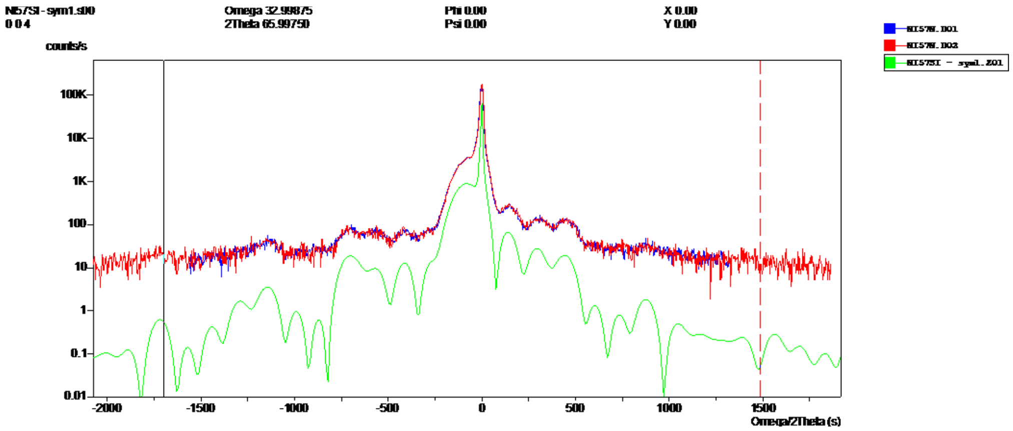


**NI58 n**

3 x InGaAsN/GaAs QW **In – 12,0%; N – 0,41%**

$d_{QW}$ – 7,0 nm

$d_{bariera}$ GaAs – 31,0 nm $Eg_{CER}$ = 1,2183 eV, $Eg_{BAC}$ = 1,2191 eV

$d_{cap}$ – 40 nm

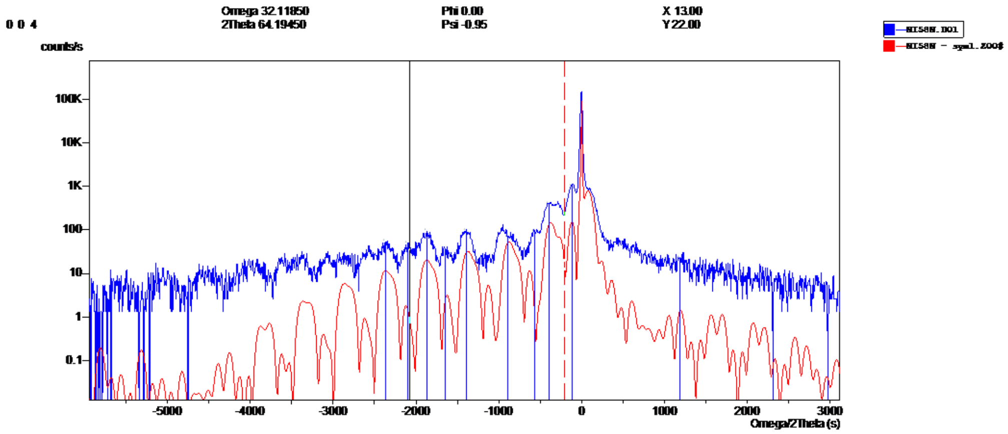

**NI59n**

3 x InGaAsN/GaAs QW **In – 15,5%;** **N – 0,29%**

$d_{QW}$ – 14 nm

$d_{bariera}$ GaAs – 37,0 nm $Eg_{CER}$ = 1,2123 eV, $Eg_{BAC}$ = 1,2171 eV

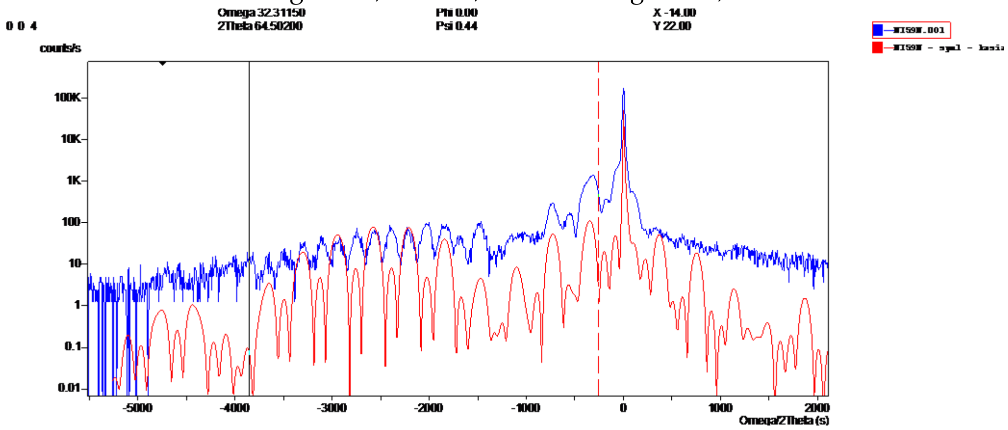


**NI60n**

3 x InGaAsN/GaAs QW **In – 6,0%;** **N – 0,33%**

$d_{QW}$ – 10,0 nm

$d_{bariera}$ GaAs – 25 nm $Eg_{CER}$ = 1,2607 eV, $Eg_{BAC}$ = 1,2608 eV

$d_{cap}$ – 38 nm

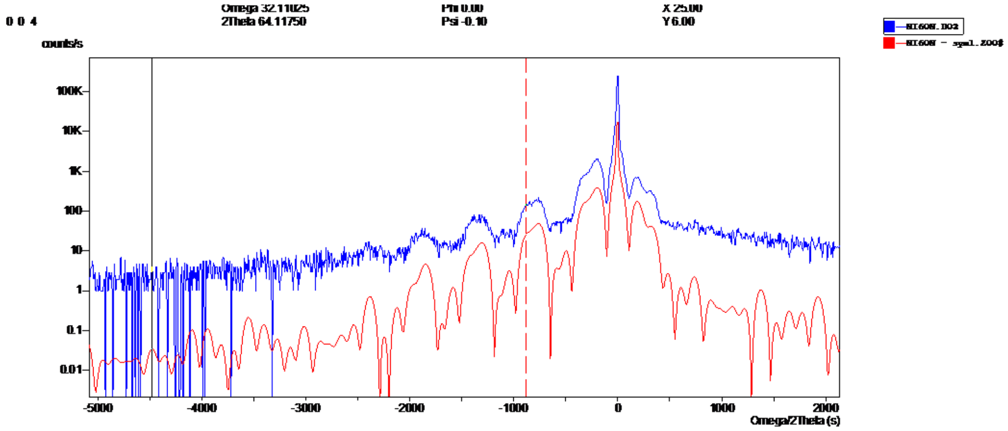


**NI61 n**

3 x InGaAsN/GaAs QW **In – 2,8%;** **N – 0,45%**

$d_{QW}$ – 10,0 nm

$d_{bariera}$ GaAs – 28 nm $Eg_{CER}$ = 1,2391 eV, $Eg_{BAC}$ = 1,2476 eV

$d_{cap}$ – 28 nm

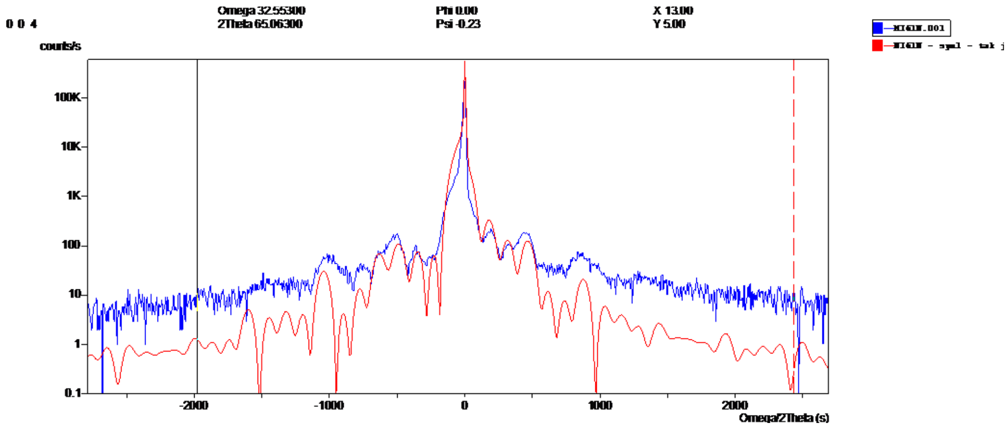


**NI65 n**

3 x InGaAsN/GaAs QW 1st layer In – 11,0% N – 0,5% d = 8,5 nm

$2^{nd}$ layer In- 21,0% N – 0,1% d = 5,6 nm

$d_{bariera}$ GaAs – 24,2 nm $Eg_{CER}$ = 1,200 eV, $Eg_{BAC}$ = 1,2029 eV

$d_{cap}$ – 32 nm

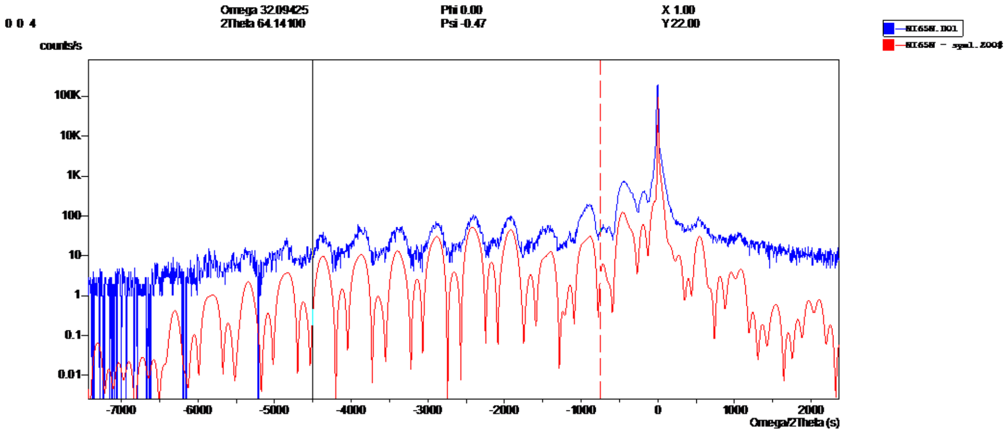


**NI66 n**

3 x InGaAsN/GaAs QW **In – 15%; N – 0,34%**

$d_{QW}$ – 13,8 nm

$d_{bariera}$ GaAs – 23,9 nm $Eg_{CER}$ = 1,212 eV, $Eg_{BAC}$ = 1,2129 eV

$d_{cap}$ – 35,7 nm

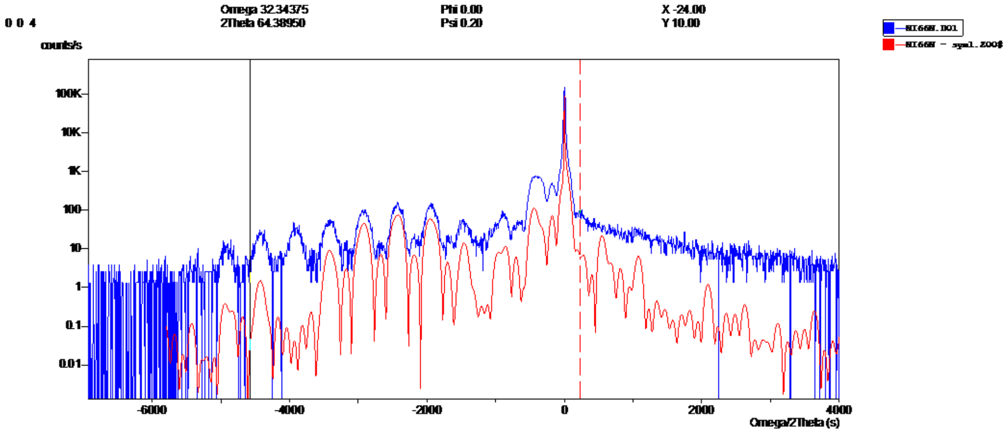

**NI67 n**

3 x InGaAsN/GaAs QW **In – 10,0%;** **N – 0,25%**

$d_{QW}$ – 10 nm

$d_{bariera}$ GaAs – 25,4 nm $Eg_{CER}$ = 1,256 eV, $Eg_{BAC}$ = 1,2576 eV

$d_{cap}$ – 32 nm

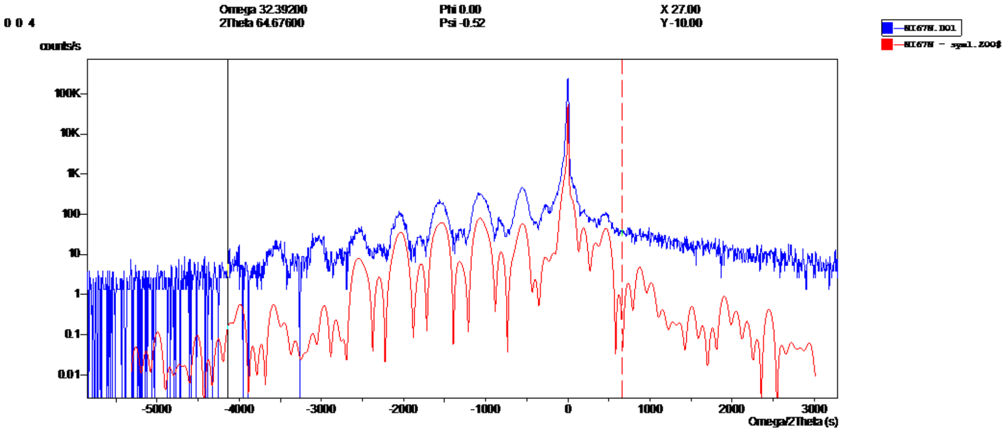


**NI68 n**

3 x

InGaAsN- **In – 16,0%;** **N – 0,24%** **d – 12,5 nm**

$d_{bariera}$ GaAs – 25,2 nm $Eg_{CER}$ = 1,22 eV, $Eg_{BAC}$ = 1,211 eV

$d_{cap}$ – 0 nm

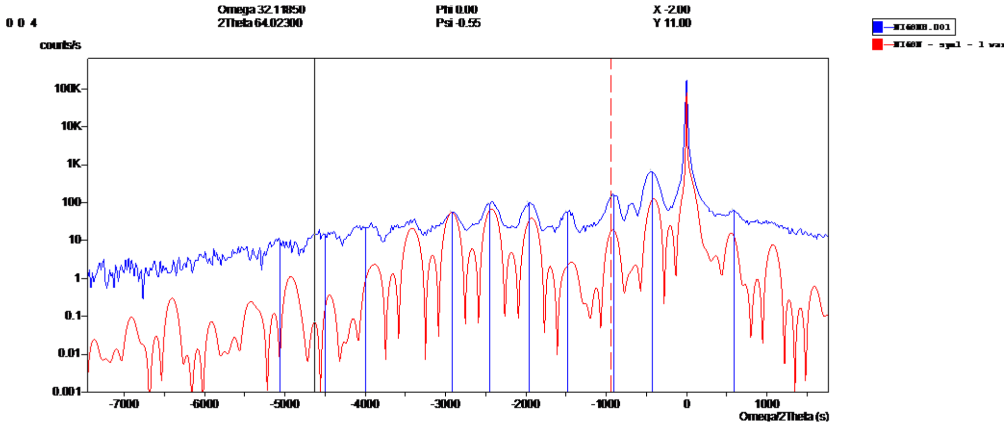


**NI69 n**

3 x InGaAsN/GaAs QW **In – 13,2%;** **N – 0,36%**

$d_{QW}$ – 15,5 nm

$d_{bariera}$ GaAs – 22,5nm $Eg_{CER}$ = 1,22 eV, $Eg_{BAC}$ = 1,22

$d_{cap}$ – 32 nm

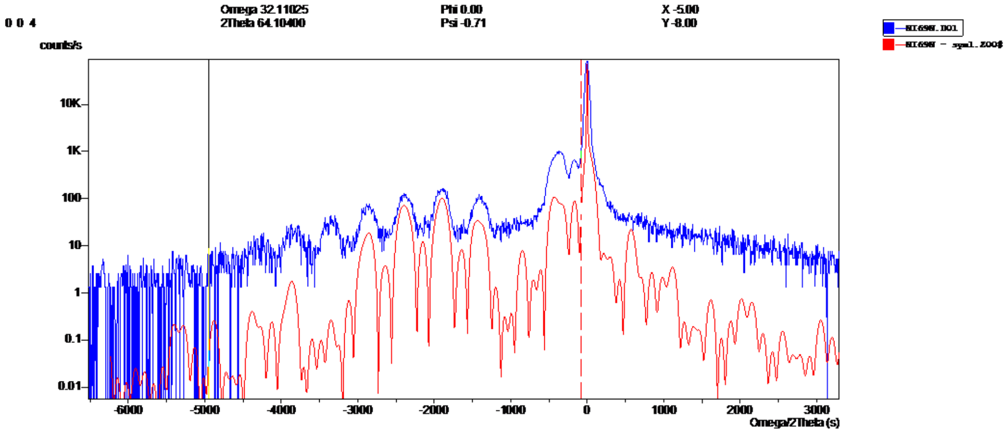


**NI70 n**

3 x InGaAsN/GaAs QW **In – 13,3%;** **N – 0,32%**

$d_{QW}$ – 15,8 nm

$d_{bariera}$ GaAs – 22,2 nm $Eg_{CER}$ = 1,225 eV, $Eg_{BAC}$ = 1,226 Ev

$d_{cap}$ – 33 nm

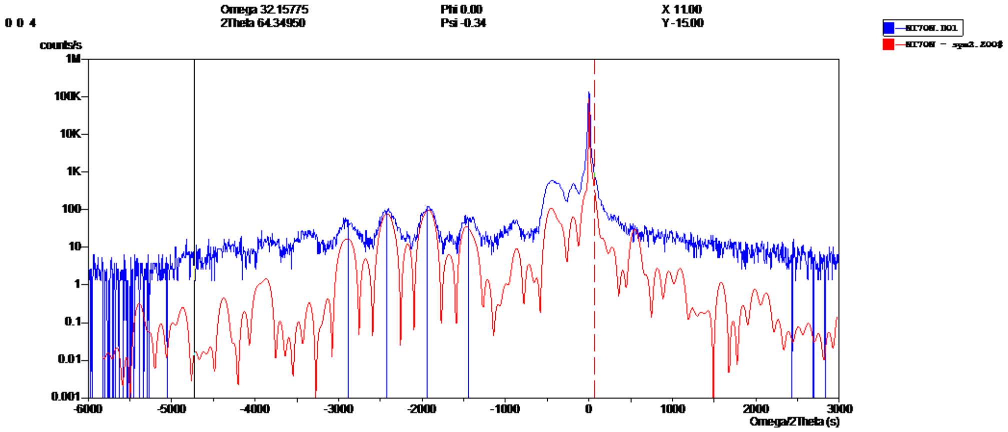

## S3. Deep-level transient spectroscopy data

### NI49n

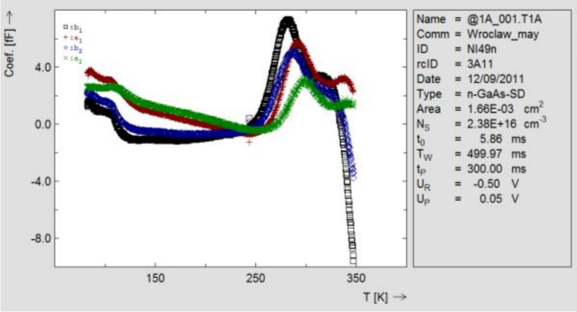


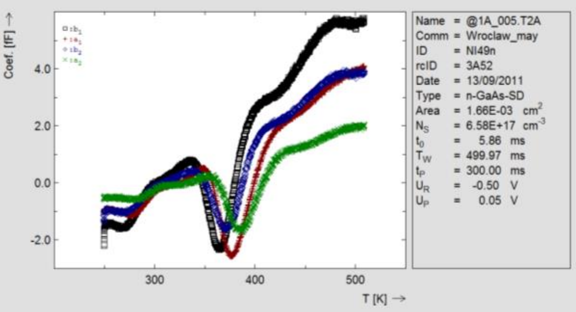


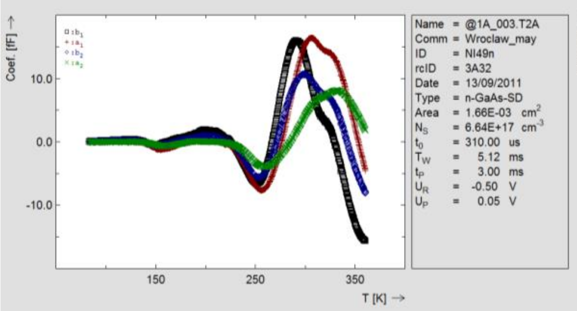


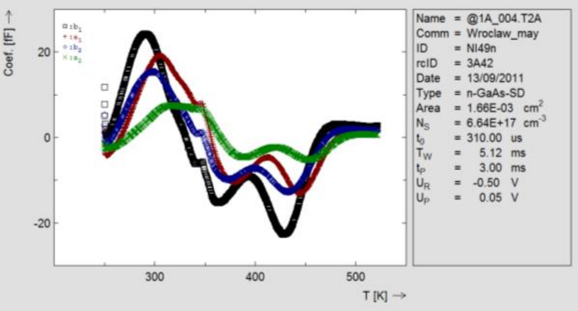


### NI51n

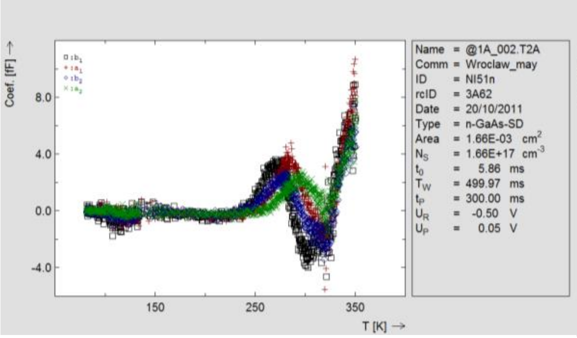


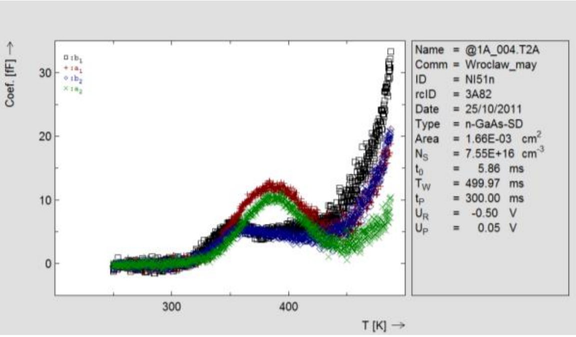


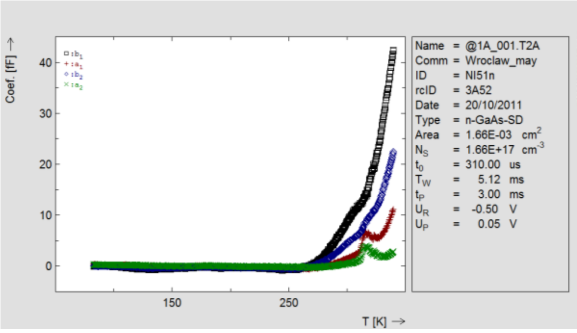


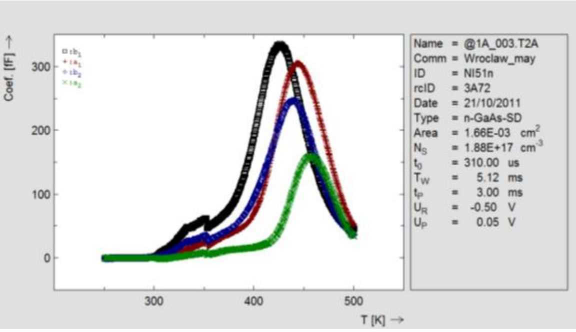

**NI55n**

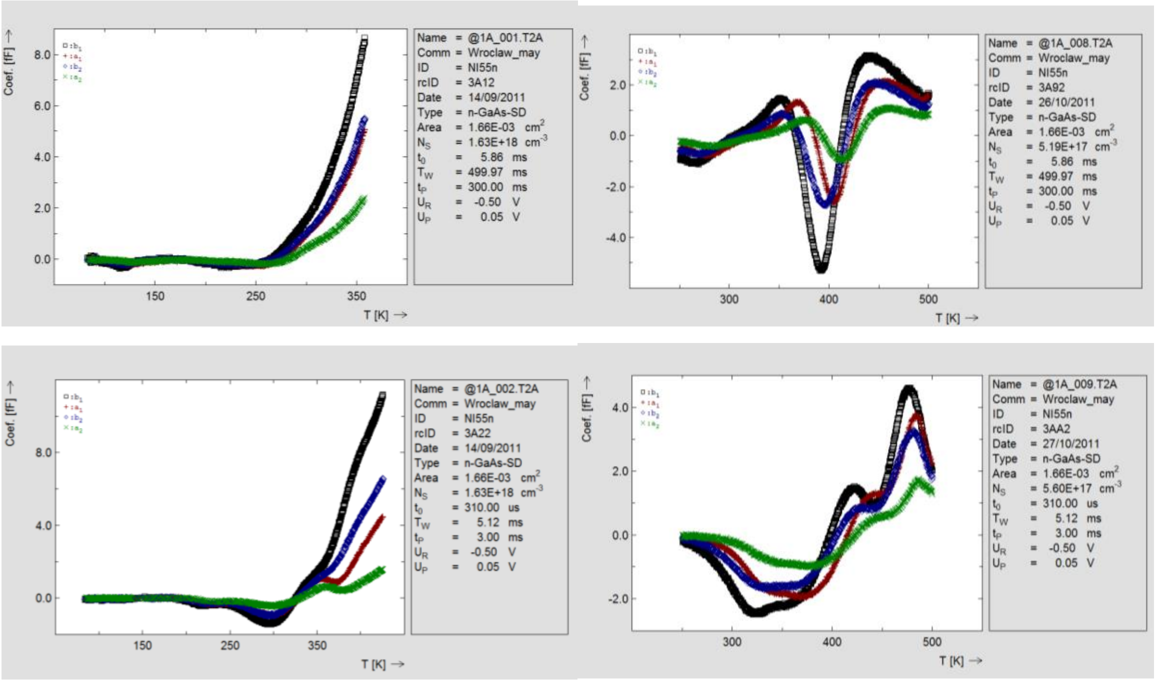
Name = @1A_001.T2A
Comm = Wroclaw_may
ID = NI55n
rcID = 3A12
Date = 14/09/2011
Type = n-GaAs-SD
Area = 1.66E-03 cm2
NS = 1.63E+18 cm-3
t0 = 5.86 ms
TW = 499.97 ms
tP = 300.00 ms
UR = -0.50 V
UP = 0.05 V
Name = @1A_008.T2A
Comm = Wroclaw_may
ID = NI55n
rcID = 3A92
Date = 26/10/2011
Type = n-GaAs-SD
Area = 1.66E-03 cm2
NS = 5.19E+17 cm-3
t0 = 5.86 ms
TW = 499.97 ms
tP = 300.00 ms
UR = -0.50 V
UP = 0.05 V
Name = @1A_002.T2A
Comm = Wroclaw_may
ID = NI55n
rcID = 3A22
Date = 14/09/2011
Type = n-GaAs-SD
Area = 1.66E-03 cm2
NS = 1.63E+18 cm-3
t0 = 310.00 us
TW = 5.12 ms
tP = 3.00 ms
UR = -0.50 V
UP = 0.05 V
Name = @1A_009.T2A
Comm = Wroclaw_may
ID = NI55n
rcID = 3AA2
Date = 27/10/2011
Type = n-GaAs-SD
Area = 1.66E-03 cm2
NS = 5.60E+17 cm-3
t0 = 310.00 us
TW = 5.12 ms
tP = 3.00 ms
UR = -0.50 V
UP = 0.05 V
Coef. [fF]
T [K]

**NI57n**

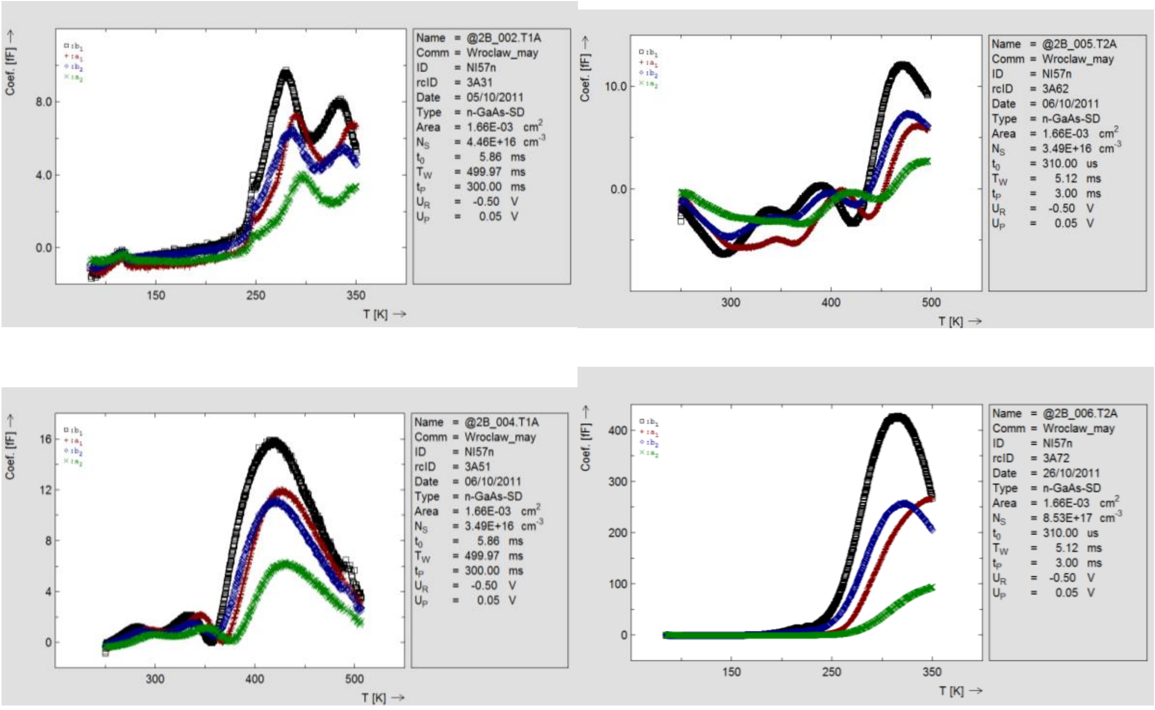
Name = @2B_002.T1A
Comm = Wroclaw_may
ID = NI57n
rcID = 3A31
Date = 05/10/2011
Type = n-GaAs-SD
Area = 1.66E-03 cm2
NS = 4.46E+16 cm-3
t0 = 5.86 ms
TW = 499.97 ms
tP = 300.00 ms
UR = -0.50 V
UP = 0.05 V
Name = @2B_005.T2A
Comm = Wroclaw_may
ID = NI57n
rcID = 3A62
Date = 06/10/2011
Type = n-GaAs-SD
Area = 1.66E-03 cm2
NS = 3.49E+16 cm-3
t0 = 310.00 us
TW = 5.12 ms
tP = 3.00 ms
UR = -0.50 V
UP = 0.05 V
Name = @2B_004.T1A
Comm = Wroclaw_may
ID = NI57n
rcID = 3A51
Date = 06/10/2011
Type = n-GaAs-SD
Area = 1.66E-03 cm2
NS = 3.49E+16 cm-3
t0 = 5.86 ms
TW = 499.97 ms
tP = 300.00 ms
UR = -0.50 V
UP = 0.05 V
Name = @2B_006.T2A
Comm = Wroclaw_may
ID = NI57n
rcID = 3A72
Date = 26/10/2011
Type = n-GaAs-SD
Area = 1.66E-03 cm2
NS = 8.53E+17 cm-3
t0 = 310.00 us
TW = 5.12 ms
tP = 3.00 ms
UR = -0.50 V
UP = 0.05 V
Coef. [fF]
T [K]

**NI58n**

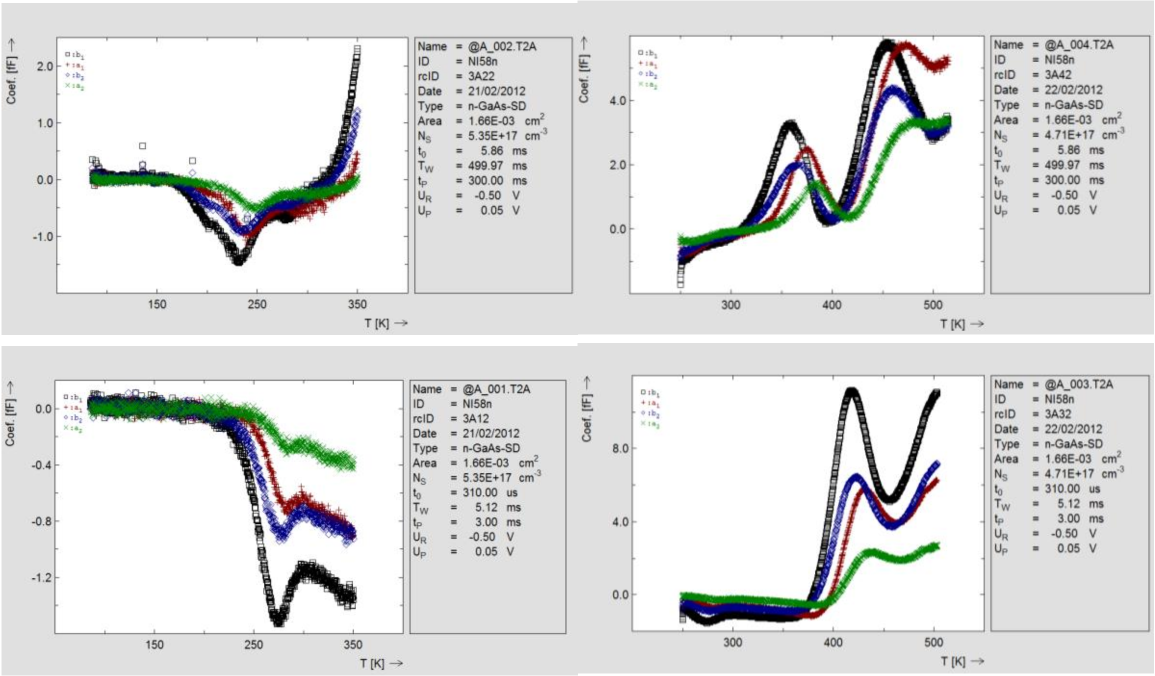
Coef. [fF] →
T [K] →
Name = @A_002.T2A
ID = NI58n
rcID = 3A22
Date = 21/02/2012
Type = n-GaAs-SD
Area = 1.66E-03 cm2
NS = 5.35E+17 cm-3
t0 = 5.86 ms
TW = 499.97 ms
tP = 300.00 ms
UR = -0.50 V
UP = 0.05 V
Name = @A_004.T2A
ID = NI58n
rcID = 3A42
Date = 22/02/2012
Type = n-GaAs-SD
Area = 1.66E-03 cm2
NS = 4.71E+17 cm-3
t0 = 5.86 ms
TW = 499.97 ms
tP = 300.00 ms
UR = -0.50 V
UP = 0.05 V
Name = @A_001.T2A
ID = NI58n
rcID = 3A12
Date = 21/02/2012
Type = n-GaAs-SD
Area = 1.66E-03 cm2
NS = 5.35E+17 cm-3
t0 = 310.00 us
TW = 5.12 ms
tP = 3.00 ms
UR = -0.50 V
UP = 0.05 V
Name = @A_003.T2A
ID = NI58n
rcID = 3A32
Date = 22/02/2012
Type = n-GaAs-SD
Area = 1.66E-03 cm2
NS = 4.71E+17 cm-3
t0 = 310.00 us
TW = 5.12 ms
tP = 3.00 ms
UR = -0.50 V
UP = 0.05 V

**NI59n**

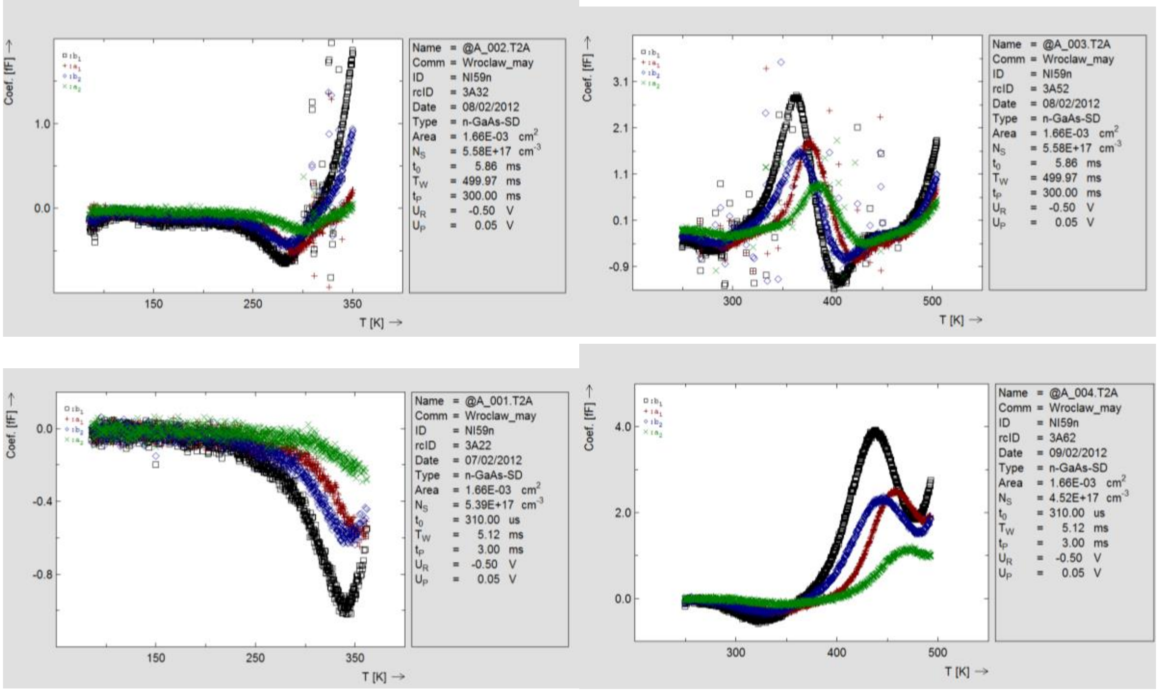
Coef. [fF] →
T [K] →
Name = @A_002.T2A
Comm = Wroclaw_may
ID = NI59n
rcID = 3A32
Date = 08/02/2012
Type = n-GaAs-SD
Area = 1.66E-03 cm2
NS = 5.58E+17 cm-3
t0 = 5.86 ms
TW = 499.97 ms
tP = 300.00 ms
UR = -0.50 V
UP = 0.05 V
Name = @A_003.T2A
Comm = Wroclaw_may
ID = NI59n
rcID = 3A52
Date = 08/02/2012
Type = n-GaAs-SD
Area = 1.66E-03 cm2
NS = 5.58E+17 cm-3
t0 = 5.86 ms
TW = 499.97 ms
tP = 300.00 ms
UR = -0.50 V
UP = 0.05 V
Name = @A_001.T2A
Comm = Wroclaw_may
ID = NI59n
rcID = 3A22
Date = 07/02/2012
Type = n-GaAs-SD
Area = 1.66E-03 cm2
NS = 5.39E+17 cm-3
t0 = 310.00 us
TW = 5.12 ms
tP = 3.00 ms
UR = -0.50 V
UP = 0.05 V
Name = @A_004.T2A
Comm = Wroclaw_may
ID = NI59n
rcID = 3A62
Date = 09/02/2012
Type = n-GaAs-SD
Area = 1.66E-03 cm2
NS = 4.52E+17 cm-3
t0 = 310.00 us
TW = 5.12 ms
tP = 3.00 ms
UR = -0.50 V
UP = 0.05 V

**NI60n**

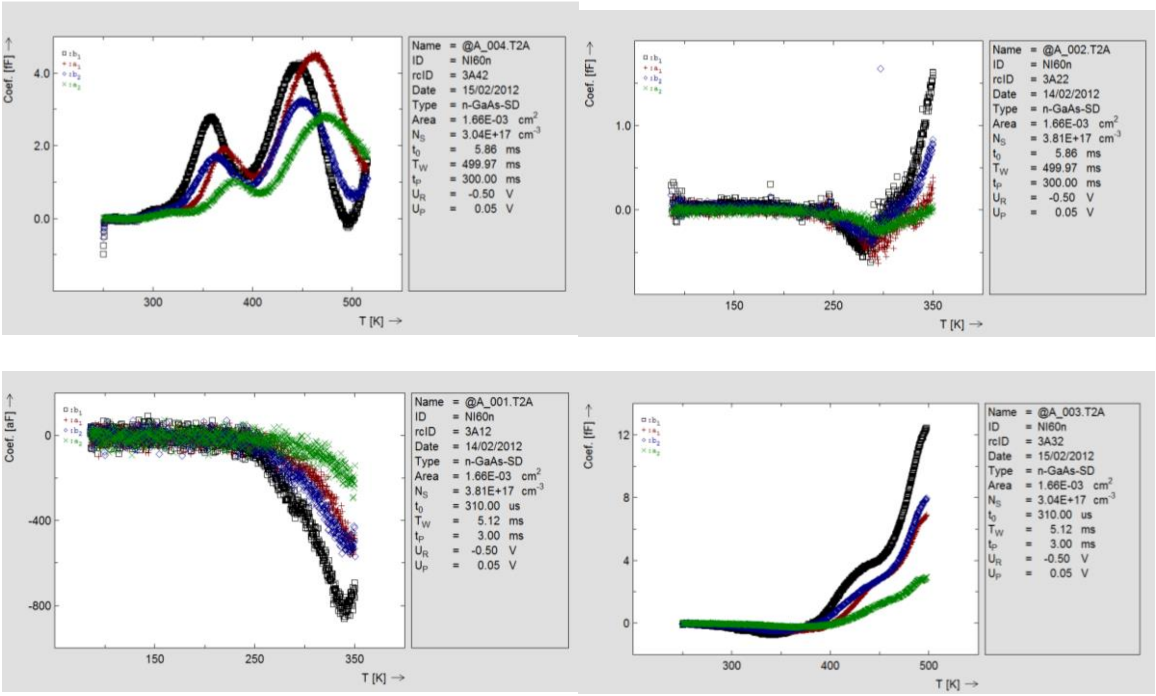
Coef. [fF]
T [K]
Name = @A_004.T2A
ID = NI60n
rcID = 3A42
Date = 15/02/2012
Type = n-GaAs-SD
Area = 1.66E-03 cm2
NS = 3.04E+17 cm-3
t0 = 5.86 ms
TW = 499.97 ms
tP = 300.00 ms
UR = -0.50 V
UP = 0.05 V
Name = @A_002.T2A
ID = NI60n
rcID = 3A22
Date = 14/02/2012
Type = n-GaAs-SD
Area = 1.66E-03 cm2
NS = 3.81E+17 cm-3
t0 = 5.86 ms
TW = 499.97 ms
tP = 300.00 ms
UR = -0.50 V
UP = 0.05 V
Coef. [aF]
Name = @A_001.T2A
ID = NI60n
rcID = 3A12
Date = 14/02/2012
Type = n-GaAs-SD
Area = 1.66E-03 cm2
NS = 3.81E+17 cm-3
t0 = 310.00 us
TW = 5.12 ms
tP = 3.00 ms
UR = -0.50 V
UP = 0.05 V
Name = @A_003.T2A
ID = NI60n
rcID = 3A32
Date = 15/02/2012
Type = n-GaAs-SD
Area = 1.66E-03 cm2
NS = 3.04E+17 cm-3
t0 = 310.00 us
TW = 5.12 ms
tP = 3.00 ms
UR = -0.50 V
UP = 0.05 V

**NI61n**

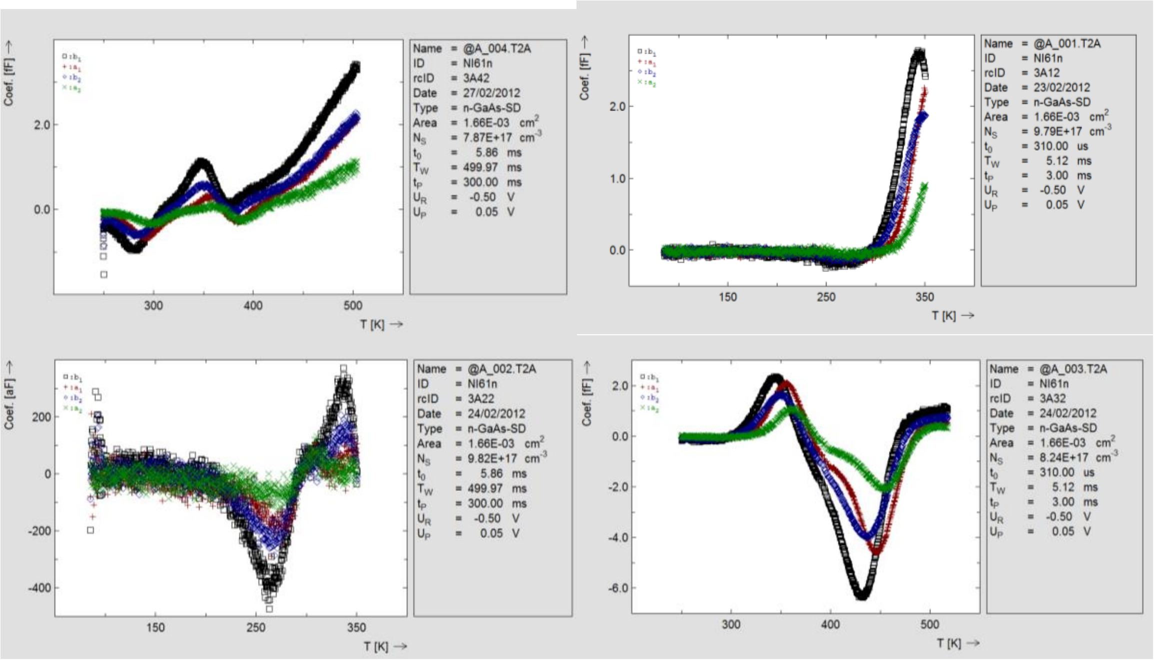
Coef. [fF]
T [K]
Name = @A_004.T2A
ID = NI61n
rcID = 3A42
Date = 27/02/2012
Type = n-GaAs-SD
Area = 1.66E-03 cm2
NS = 7.87E+17 cm-3
t0 = 5.86 ms
TW = 499.97 ms
tP = 300.00 ms
UR = -0.50 V
UP = 0.05 V
Name = @A_001.T2A
ID = NI61n
rcID = 3A12
Date = 23/02/2012
Type = n-GaAs-SD
Area = 1.66E-03 cm2
NS = 9.79E+17 cm-3
t0 = 310.00 us
TW = 5.12 ms
tP = 3.00 ms
UR = -0.50 V
UP = 0.05 V
Coef. [aF]
Name = @A_002.T2A
ID = NI61n
rcID = 3A22
Date = 24/02/2012
Type = n-GaAs-SD
Area = 1.66E-03 cm2
NS = 9.82E+17 cm-3
t0 = 5.86 ms
TW = 499.97 ms
tP = 300.00 ms
UR = -0.50 V
UP = 0.05 V
Name = @A_003.T2A
ID = NI61n
rcID = 3A32
Date = 24/02/2012
Type = n-GaAs-SD
Area = 1.66E-03 cm2
NS = 8.24E+17 cm-3
t0 = 310.00 us
TW = 5.12 ms
tP = 3.00 ms
UR = -0.50 V
UP = 0.05 V

**NI65n**

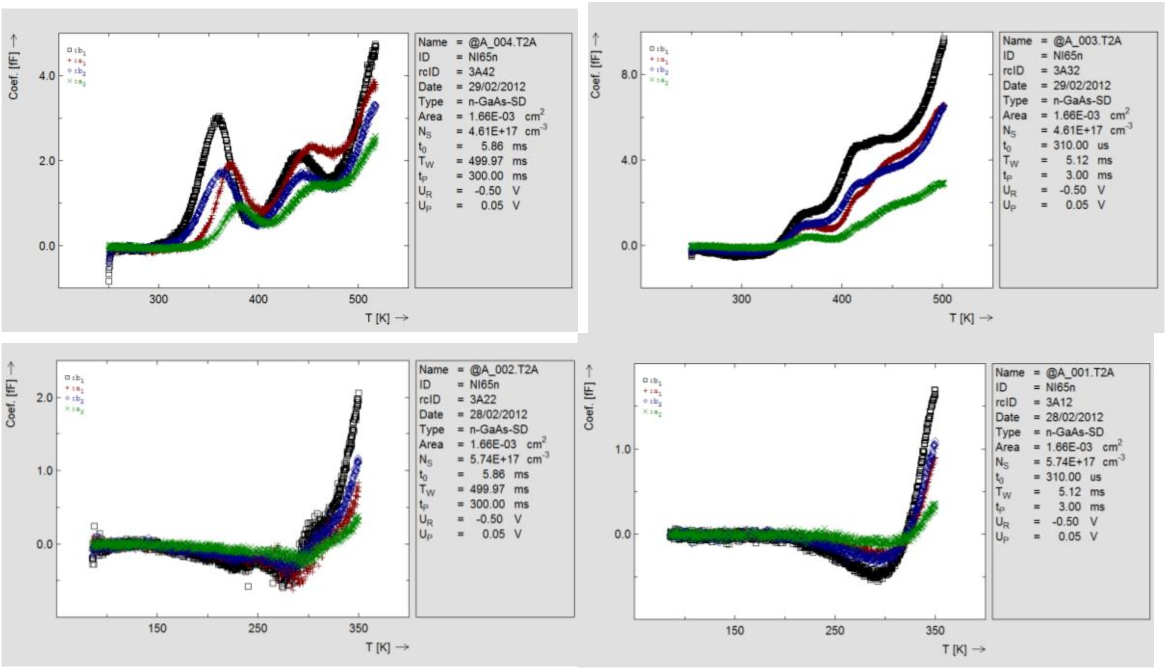
Coef. [fF] →
T [K] →
Name = @A_004.T2A
ID = NI65n
rcID = 3A42
Date = 29/02/2012
Type = n-GaAs-SD
Area = 1.66E-03 cm2
NS = 4.61E+17 cm-3
t0 = 5.86 ms
TW = 499.97 ms
tP = 300.00 ms
UR = -0.50 V
UP = 0.05 V
Name = @A_003.T2A
ID = NI65n
rcID = 3A32
Date = 29/02/2012
Type = n-GaAs-SD
Area = 1.66E-03 cm2
NS = 4.61E+17 cm-3
t0 = 310.00 us
TW = 5.12 ms
tP = 3.00 ms
UR = -0.50 V
UP = 0.05 V
Name = @A_002.T2A
ID = NI65n
rcID = 3A22
Date = 28/02/2012
Type = n-GaAs-SD
Area = 1.66E-03 cm2
NS = 5.74E+17 cm-3
t0 = 5.86 ms
TW = 499.97 ms
tP = 300.00 ms
UR = -0.50 V
UP = 0.05 V
Name = @A_001.T2A
ID = NI65n
rcID = 3A12
Date = 28/02/2012
Type = n-GaAs-SD
Area = 1.66E-03 cm2
NS = 5.74E+17 cm-3
t0 = 310.00 us
TW = 5.12 ms
tP = 3.00 ms
UR = -0.50 V
UP = 0.05 V

**NI66n**

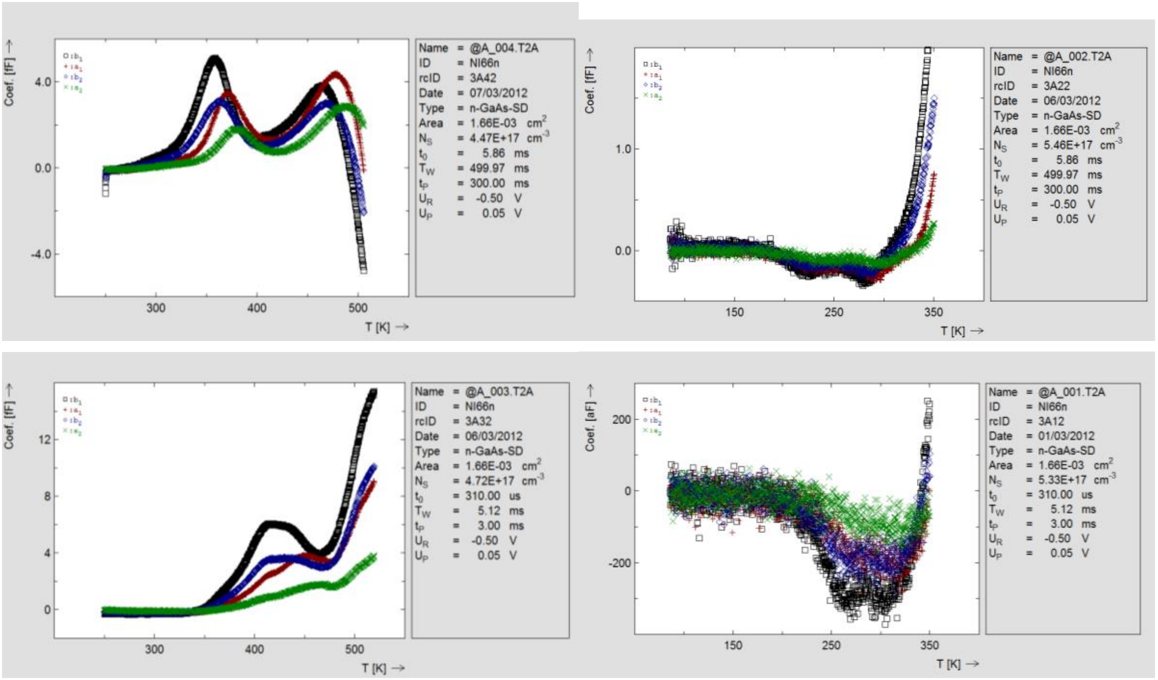
Coef. [fF] →
Coef. [aF] →
T [K] →
Name = @A_004.T2A
ID = NI66n
rcID = 3A42
Date = 07/03/2012
Type = n-GaAs-SD
Area = 1.66E-03 cm2
NS = 4.47E+17 cm-3
t0 = 5.86 ms
TW = 499.97 ms
tP = 300.00 ms
UR = -0.50 V
UP = 0.05 V
Name = @A_002.T2A
ID = NI66n
rcID = 3A22
Date = 06/03/2012
Type = n-GaAs-SD
Area = 1.66E-03 cm2
NS = 5.46E+17 cm-3
t0 = 5.86 ms
TW = 499.97 ms
tP = 300.00 ms
UR = -0.50 V
UP = 0.05 V
Name = @A_003.T2A
ID = NI66n
rcID = 3A32
Date = 06/03/2012
Type = n-GaAs-SD
Area = 1.66E-03 cm2
NS = 4.72E+17 cm-3
t0 = 310.00 us
TW = 5.12 ms
tP = 3.00 ms
UR = -0.50 V
UP = 0.05 V
Name = @A_001.T2A
ID = NI66n
rcID = 3A12
Date = 01/03/2012
Type = n-GaAs-SD
Area = 1.66E-03 cm2
NS = 5.33E+17 cm-3
t0 = 310.00 us
TW = 5.12 ms
tP = 3.00 ms
UR = -0.50 V
UP = 0.05 V

## NI67n

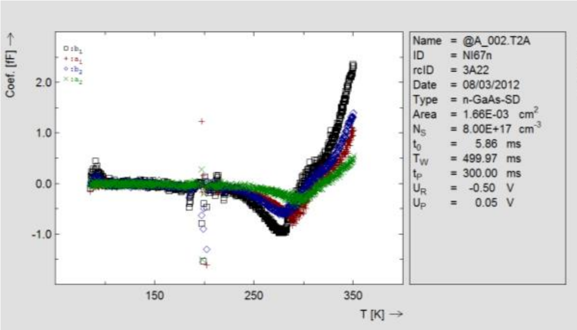
Coef. [fF] →
T [K] →
Name = @A_002.T2A
ID = NI67n
rcID = 3A22
Date = 08/03/2012
Type = n-GaAs-SD
Area = 1.66E-03 cm$^2$
$N_S$ = 8.00E+17 cm$^{-3}$
$t_0$ = 5.86 ms
$T_W$ = 499.97 ms
$t_P$ = 300.00 ms
$U_R$ = -0.50 V
$U_P$ = 0.05 V

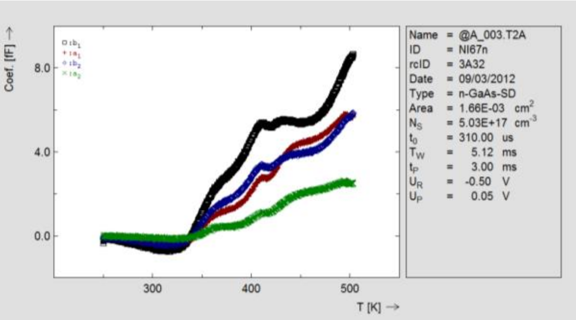
Coef. [fF] →
T [K] →
Name = @A_003.T2A
ID = NI67n
rcID = 3A32
Date = 09/03/2012
Type = n-GaAs-SD
Area = 1.66E-03 cm$^2$
$N_S$ = 5.03E+17 cm$^{-3}$
$t_0$ = 310.00 us
$T_W$ = 5.12 ms
$t_P$ = 3.00 ms
$U_R$ = -0.50 V
$U_P$ = 0.05 V

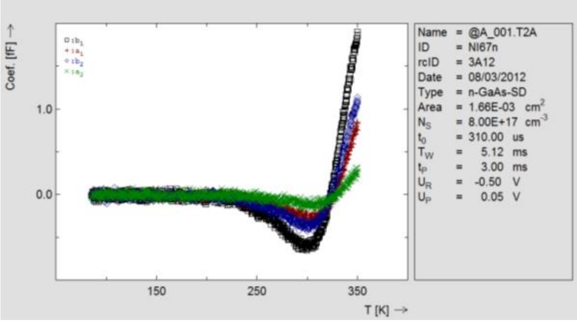
Coef. [fF] →
T [K] →
Name = @A_001.T2A
ID = NI67n
rcID = 3A12
Date = 08/03/2012
Type = n-GaAs-SD
Area = 1.66E-03 cm$^2$
$N_S$ = 8.00E+17 cm$^{-3}$
$t_0$ = 310.00 us
$T_W$ = 5.12 ms
$t_P$ = 3.00 ms
$U_R$ = -0.50 V
$U_P$ = 0.05 V

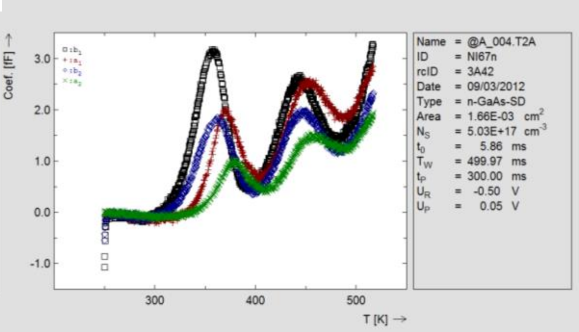
Coef. [fF] →
T [K] →
Name = @A_004.T2A
ID = NI67n
rcID = 3A42
Date = 09/03/2012
Type = n-GaAs-SD
Area = 1.66E-03 cm$^2$
$N_S$ = 5.03E+17 cm$^{-3}$
$t_0$ = 5.86 ms
$T_W$ = 499.97 ms
$t_P$ = 300.00 ms
$U_R$ = -0.50 V
$U_P$ = 0.05 V

## NI68n

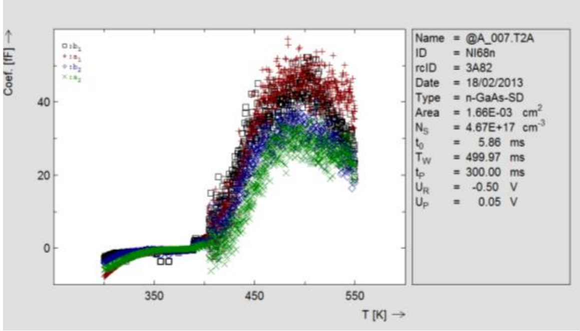
Coef. [fF] →
T [K] →
Name = @A_007.T2A
ID = NI68n
rcID = 3A82
Date = 18/02/2013
Type = n-GaAs-SD
Area = 1.66E-03 cm$^2$
$N_S$ = 4.67E+17 cm$^{-3}$
$t_0$ = 5.86 ms
$T_W$ = 499.97 ms
$t_P$ = 300.00 ms
$U_R$ = -0.50 V
$U_P$ = 0.05 V

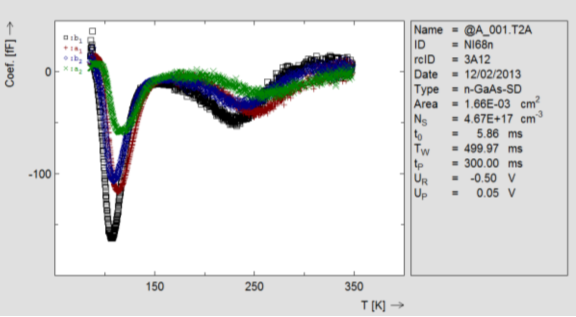
Coef. [fF] →
T [K] →
Name = @A_001.T2A
ID = NI68n
rcID = 3A12
Date = 12/02/2013
Type = n-GaAs-SD
Area = 1.66E-03 cm$^2$
$N_S$ = 4.67E+17 cm$^{-3}$
$t_0$ = 5.86 ms
$T_W$ = 499.97 ms
$t_P$ = 300.00 ms
$U_R$ = -0.50 V
$U_P$ = 0.05 V

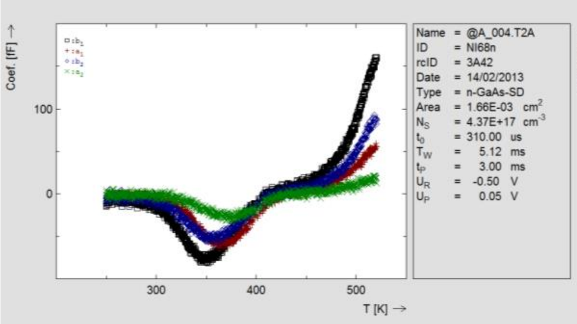
Coef. [fF] →
T [K] →
Name = @A_004.T2A
ID = NI68n
rcID = 3A42
Date = 14/02/2013
Type = n-GaAs-SD
Area = 1.66E-03 cm$^2$
$N_S$ = 4.37E+17 cm$^{-3}$
$t_0$ = 310.00 us
$T_W$ = 5.12 ms
$t_P$ = 3.00 ms
$U_R$ = -0.50 V
$U_P$ = 0.05 V

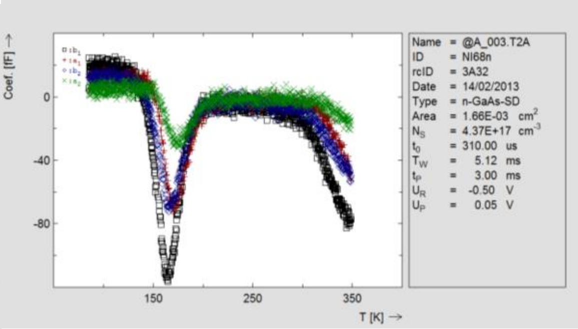
Coef. [fF] →
T [K] →
Name = @A_003.T2A
ID = NI68n
rcID = 3A32
Date = 14/02/2013
Type = n-GaAs-SD
Area = 1.66E-03 cm$^2$
$N_S$ = 4.37E+17 cm$^{-3}$
$t_0$ = 310.00 us
$T_W$ = 5.12 ms
$t_P$ = 3.00 ms
$U_R$ = -0.50 V
$U_P$ = 0.05 V

**NI69n**

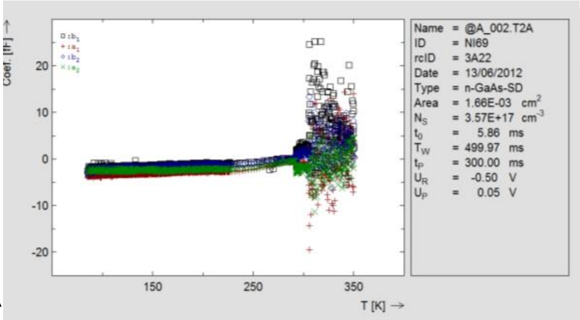
Coef. [fF] →
T [K] →
Name = @A_002.T2A
ID = NI69
rcID = 3A22
Date = 13/06/2012
Type = n-GaAs-SD
Area = 1.66E-03 cm$^{2}$
$N_S$ = 3.57E+17 cm$^{-3}$
$t_0$ = 5.86 ms
$T_W$ = 499.97 ms
$t_P$ = 300.00 ms
$U_R$ = -0.50 V
$U_P$ = 0.05 V

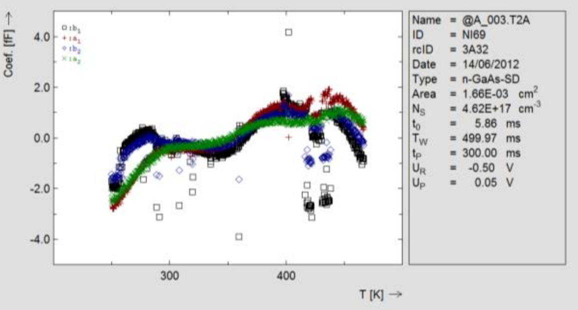
Coef. [fF] →
T [K] →
Name = @A_003.T2A
ID = NI69
rcID = 3A32
Date = 14/06/2012
Type = n-GaAs-SD
Area = 1.66E-03 cm$^{2}$
$N_S$ = 4.62E+17 cm$^{-3}$
$t_0$ = 5.86 ms
$T_W$ = 499.97 ms
$t_P$ = 300.00 ms
$U_R$ = -0.50 V
$U_P$ = 0.05 V

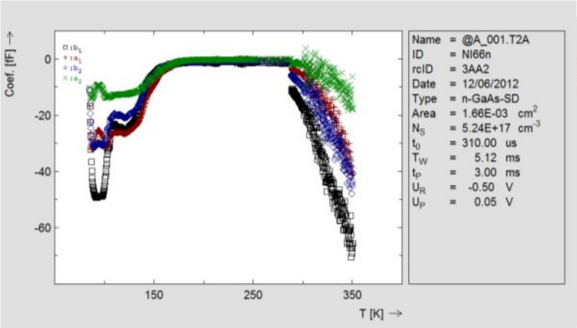
Coef. [fF] →
T [K] →
Name = @A_001.T2A
ID = NI66n
rcID = 3AA2
Date = 12/06/2012
Type = n-GaAs-SD
Area = 1.66E-03 cm$^{2}$
$N_S$ = 5.24E+17 cm$^{-3}$
$t_0$ = 310.00 us
$T_W$ = 5.12 ms
$t_P$ = 3.00 ms
$U_R$ = -0.50 V
$U_P$ = 0.05 V

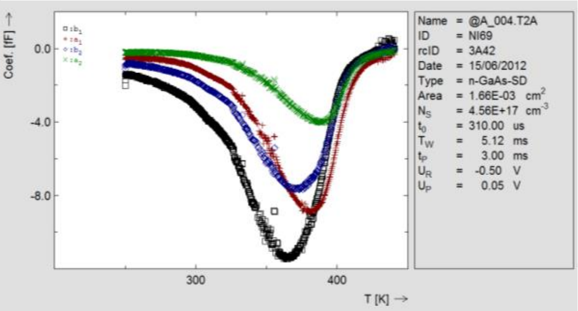
Coef. [fF] →
T [K] →
Name = @A_004.T2A
ID = NI69
rcID = 3A42
Date = 15/06/2012
Type = n-GaAs-SD
Area = 1.66E-03 cm$^{2}$
$N_S$ = 4.56E+17 cm$^{-3}$
$t_0$ = 310.00 us
$T_W$ = 5.12 ms
$t_P$ = 3.00 ms
$U_R$ = -0.50 V
$U_P$ = 0.05 V

**NI70n**

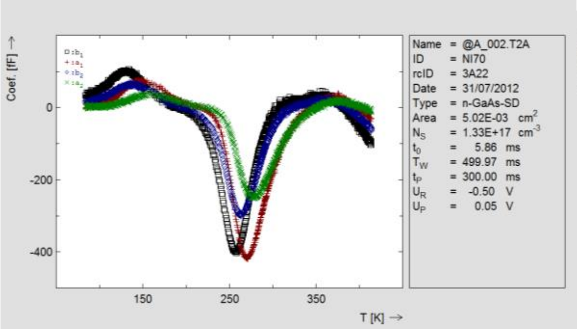
Coef. [fF] →
T [K] →
Name = @A_002.T2A
ID = NI70
rcID = 3A22
Date = 31/07/2012
Type = n-GaAs-SD
Area = 5.02E-03 cm$^{2}$
$N_S$ = 1.33E+17 cm$^{-3}$
$t_0$ = 5.86 ms
$T_W$ = 499.97 ms
$t_P$ = 300.00 ms
$U_R$ = -0.50 V
$U_P$ = 0.05 V

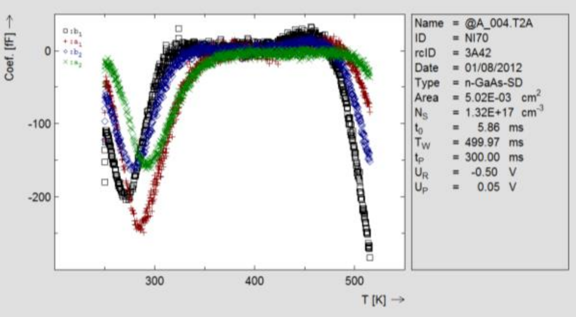
Coef. [fF] →
T [K] →
Name = @A_004.T2A
ID = NI70
rcID = 3A42
Date = 01/08/2012
Type = n-GaAs-SD
Area = 5.02E-03 cm$^{2}$
$N_S$ = 1.32E+17 cm$^{-3}$
$t_0$ = 5.86 ms
$T_W$ = 499.97 ms
$t_P$ = 300.00 ms
$U_R$ = -0.50 V
$U_P$ = 0.05 V

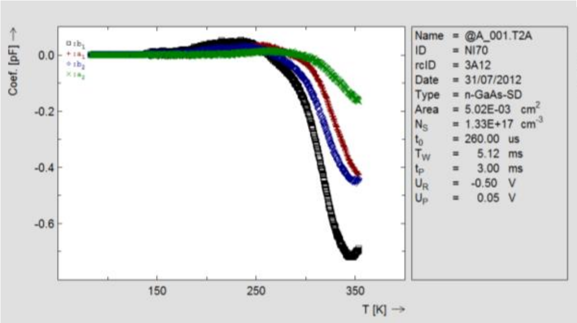
Coef. [pF] →
T [K] →
Name = @A_001.T2A
ID = NI70
rcID = 3A12
Date = 31/07/2012
Type = n-GaAs-SD
Area = 5.02E-03 cm$^{2}$
$N_S$ = 1.33E+17 cm$^{-3}$
$t_0$ = 260.00 us
$T_W$ = 5.12 ms
$t_P$ = 3.00 ms
$U_R$ = -0.50 V
$U_P$ = 0.05 V

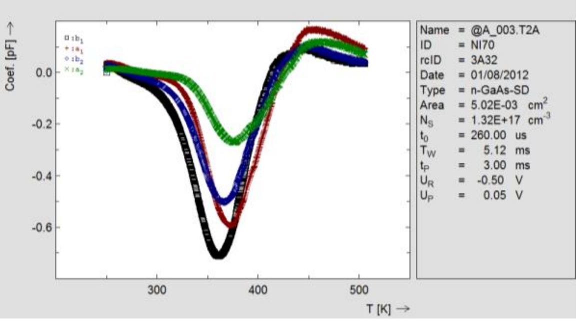
Coef. [pF] →
T [K] →
Name = @A_003.T2A
ID = NI70
rcID = 3A32
Date = 01/08/2012
Type = n-GaAs-SD
Area = 5.02E-03 cm$^{2}$
$N_S$ = 1.32E+17 cm$^{-3}$
$t_0$ = 260.00 us
$T_W$ = 5.12 ms
$t_P$ = 3.00 ms
$U_R$ = -0.50 V
$U_P$ = 0.05 V

## I218n

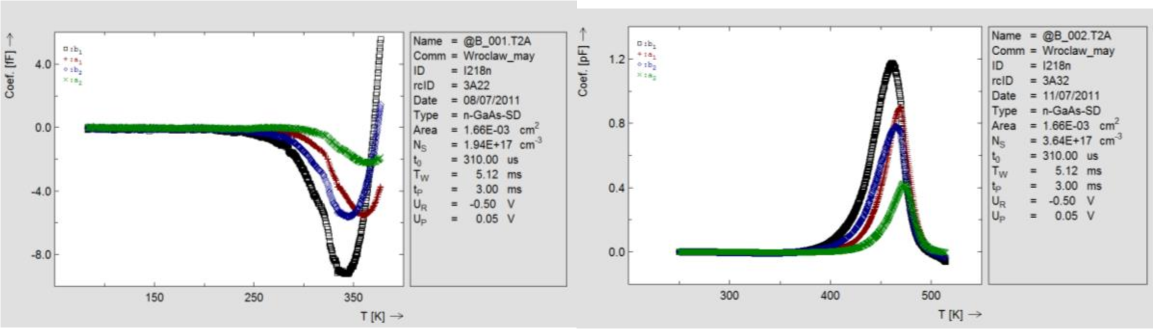
Coef. [fF] →
4.0
0.0
-4.0
-8.0
150
250
350
T [K] →
□ :b1
+ :a1
○ :b2
× :a2
Name = @B_001.T2A
Comm = Wroclaw_may
ID = I218n
rcID = 3A22
Date = 08/07/2011
Type = n-GaAs-SD
Area = 1.66E-03 cm2
NS = 1.94E+17 cm-3
t0 = 310.00 us
TW = 5.12 ms
tP = 3.00 ms
UR = -0.50 V
UP = 0.05 V
Coef. [pF] →
1.2
0.8
0.4
0.0
300
400
500
T [K] →
□ :b1
+ :a1
○ :b2
× :a2
Name = @B_002.T2A
Comm = Wroclaw_may
ID = I218n
rcID = 3A32
Date = 11/07/2011
Type = n-GaAs-SD
Area = 1.66E-03 cm2
NS = 3.64E+17 cm-3
t0 = 310.00 us
TW = 5.12 ms
tP = 3.00 ms
UR = -0.50 V
UP = 0.05 V
